# A Reflection on the Main Ethical Obstacles Related to the Strategic Action “*O Brasil Conta Comigo*”

**DOI:** 10.3389/fpsyt.2021.619296

**Published:** 2021-02-12

**Authors:** Jucycler Ferreira Freitas, Jucier Gonçalves Júnior, Estelita Lima Cândido

**Affiliations:** ^1^Programa de Pós-Graduação em Desenvolvimento Regional Sustentável – PRODER, Universidade Federal Do Cariri (UFCA), Juazeiro Do Norte, Brazil; ^2^Department of Internal Medicine, Santa Casa de Misericórdia, Fortaleza, Brazil

**Keywords:** bioethics, coronavirus infection, public health, education, medical, government program

## Introduction

The COVID-19 pandemic began in China in December 2019 and quickly spread to the rest of the world, forcing countries to develop strategies to fight the disease. By January 16, 2021, the virus had infected 91,816,091 people and caused 1,986,871 deaths (1).

Countries have adopted policies requiring individuals to practice social isolation, enforced through administrative and criminal sanctions. Additionally, it was necessary to create measures that would increase the number of professionals available to serve the population in medical and hospital units (2–5).

Countries such as the United Kingdom (2), Denmark (3), and Brazil (4, 5) adopted measures capable of having a direct impact on academic training and subsequently on the professional lives of students in the health field, such as accelerating medical graduation and the recruitment of students from other disciplines, such as nursing and pharmacy to act on the front lines of the pandemic response. To attract volunteer students from other areas of health, the government proposals offer benefits ranging from financial resources to advantages over other students in public tenders. These questions and others that will be raised in this paper represent ethical conflicts that need to be discussed from an academic, occupational, and human perspective.

This paper aims to promote a reflection on the main ethical obstacles related to the Strategic Action “*O Brasil Conta Comigo*” (“Brazil counts on me”), which is focused on employing students of health courses to face the coronavirus pandemic (COVID-19). To this end, our arguments are based on the strategy itself, on Notice 4/2020, on Brazillian laws, and on ordinances issued by official bodies, including the Ministry of Health of Brazil (DH), the Ministry of Education (ME), and the Open University of the Brazilian Unified Health System (UNA/SUS).

## Discussion

### A Brief Presentation of the Program “O Brasil Conta Comigo” (Brazil Counts on Me)

The Strategic Action “*O Brasil Conta Comigo*” (“Brazil counts on me”) consists of an emergency measure implemented by the Brazilian government, in the form of a program with national coverage, whose objective is to optimize the availability of health services within the scope of SUS in order to contain the COVID-19 pandemic. The aforementioned strategic action was instituted by Ordinance numbers 356, of March 20, 2020 (4) and 492, of March 23, 2020 (5), which originated respectively from the MH and the DH. This program will be achieved through the participation of undergraduate students in healthcare delivery, while the state of public health emergency resulting from the worldwide pandemic persists (5).

The strategic action is aimed at students regularly enrolled in the last 2 years of the medical course, and in the last year of the nursing, pharmacy and physiotherapy courses of the federal education system (4). Normally, these health courses have, prior to graduation, an internship regulated and supervised by qualified teachers who belong to the higher education institutions to which the students belong. In the “O Brasil Conta Comigo” program these students act in care, that is, face-to-face activities for patients with or without suspected COVID-19 in health units during the pandemic (5) under the supervision of local professionals. During the participation of students, they will be involved in activities including (a) screening patients; (b) outpatient care; (c) hospital care; and (d) care in the home care system in internal medicine, primary health care, and pediatrics. There is a pre-established workload (20–40 h) and the proposal provides for professor / preceptor supervision accredited by UNA/SUS (5–7) for theoretical activities. Thus, the program fits into an internship modality.

The participation of students can be done in two ways: computing the workload in the program as the workload of the supervised internship; or through voluntary work. There are three compensations provided for in the Public Notice: (I) an additional 10% in the public selection process for Health Residency Programs promoted by the Ministry of Health; (II) obtaining a discount on the monthly fee, to be defined and granted by Higher Education Institutions; or (III) receiving a scholarship to work in SUS (4, 5).

It is up to the managers and personnel responsible for the health units of the municipalities, states, and the Federal District, to adhere to the strategic action, to select the advisors for the students and to determine the number of places destined for the students.

Thus, medical, nursing, pharmacy, and physical therapy students, in supplementing the health service, will face the coronavirus and guarantee additional healthcare to the population (6, 7). The strategic action serves as a mechanism for optimizing the availability of services in the Unified Health System.

### Ethical Implications of the “O Brasil Conta Comigo” Program (Brazil Counts on Me)

First, the educational system and society need to reflect on the risks and benefits of exposing students to the attempt at trial and error in the face of ethical situations distanced from controlled teaching environments (8). It is necessary to remember that in extreme situations and when there is a lack of adequate information such as the pandemic, decision making involves and requires much more from the professional. In this way, ethical decision-making becomes quite challenging for young professionals who have not yet completed their undergraduate studies. In this context, good teacher preceptorship / assistance is essential (8). Although the “O Brasil Conta Comigo” Program provides for the supervision of students by local professionals linked to UNSA / SUS, it cannot guarantee their supervision by professionals who are qualified to teach or who are linked to educational institutions. Also, it does not establish assessment instruments to verify the teaching-learning process (5). Further, the workload is excessive, based on the assumption that the program constitutes an internship (6).

These aspects differ from Law number 11,788, of September 25, 2008—Internship Law (7) in several points, such as student supervision, monitoring criteria, workload requirements, and security measures. The aforementioned law requires the guidance of a professor in the area indicated by the educational institution. This implies closer monitoring of students through the registration of attendance, and evaluation according to the rules of the student's home institution. It also provides for the internship to be carried out with a maximum workload of 30 h per week and a personal accident insurance agreement in favor of the students.

Secondly, it is noteworthy that this proposal to insert students in the fight against COVID-19 in health services arose while universities suspended face-to-face classes and the internship fields suspended their activities, no longer receiving students. The reasons for this suspension included the risk of infection, and the logistical difficulties inherent in ensuring the supervision of students. Given the pandemic period and its severity, the Brazilian Medical Association (9), before October 2, 2020, received 3,931 complaints about the absence of PPE, which affects 782 municipalities and mainly concerns the absence of masks, goggles, and waterproof covers. In this sense, according to the normative acts that govern the Strategic Action “O Brasil Conta Comigo” (“Brazil counts on me”), it is up to the federated entities to provide PPE to their students. Thus, there must be an increase in PPE transfers, given that several municipalities do not even have sufficient materials for their employees.

In this context, it is important to remember that in Brazil, SUS has an important shortage throughout the national territory of personal protective equipment (PPE) (9), of qualified professionals, and services prepared for teaching-learning activities (10). An example of this is that, according to Gonçalves Júnior et al. the shortage of professionals with an adequate profile for integral care, coupled with insufficiency and maldistribution, are some of the main barriers to the universalization of access to health in Brazil, where the number of physicians per inhabitant (2.11 doctors/1,000 inhabitants) is small compared to other countries such as France (3.0 physicians/1,000 inhabitants), the United Kingdom (2.7 physicians/1,000 inhabitants), and Sweden (4.0 physicians/1,000 inhabitants) (11). The “O Brasil Conta Comigo” program does not take this perspective into account. Thus, there is a concern that these students are being placed in extreme situations, without sufficient scientific knowledge, with inadequate preceptorship, and lacking the minimum necessary PPE.

Thirdly, it can be seen that the aforementioned strategic action, through the notice 4/2020, does not include insurance against accidents and recess, as required in the internship law. According to the Pan American Health Organization (9), almost 570 thousand health professionals were infected and more than 2,500 succumbed to the virus. Of these, 258,200 infected health professionals are from Brazil, accounting for 226 deaths prior to August of 2020 (12).

Fourth, another perspective to be explored is the impact that these experiences can have on students' mental health. O'Byrne et al. (13) emphasize that those who engage in such work without sufficient preparation are subject to moral trauma and adverse health outcomes. Brazilian medical school interns were asked about whether they felt prepared to act in the fight against the pandemic, and 57.5% stated that they did not (14). Among Spanish students in Nursing and Medicine courses, a survey revealed a lack of knowledge regarding virus transmission and basic preventive measures, and further revealed that a low percentage of students had received training specific thereto (15). According to Rolim Neto et al. (16), Work-related stress is a potential cause of concern for health professionals. It has been associated with anxiety including multiple clinical cases thereof, depression in the face of the coexistence with countless deaths, and long work shifts that incorporate the most diverse unknowns and heightened demands in the treatment of patients with COVID-19. Therefore, it is an important indicator of psychic exhaustion. Besides that, situations of extreme vulnerability such as the pandemic resonate with health professionals who suffer or have suffered from anxiety and obsessive-compulsive disorder (OCD) in the treatment of patients in hospitals. Panic attacks can also be a response to the stress load linked to the demands imposed by the coronavirus outbreak (16). Considering that experienced professionals become sick psychically in unhealthy environments such as the pandemic, it can be expected that the impact of the pandemic on students' mental health will be even more severe.

Fifthly, we want to discuss the tempting benefits or advantages offered to recruits, such as the receipt of a scholarship and of a certificate that guarantee such students an additional score of 10% in the public admission process for residency programs promoted by the DH, which is valid for 2 years, counting from the date of certificate issue (5, 6). This benefit violates the principle of isonomy, since many students who are prevented from participating in the process because they form part of an at-risk group, for instance, will be penalized in the aforementioned process. The Brazilian Medical Education Association (17) also disagrees with this point and warns that interns who have already completed the rotations in the areas highlighted in Ordinance No. 492 and Request for Proposal No. 4 will not have the opportunity to participate. In the competitive health market, offering bonuses in public tenders, such as the residency program, would encourage young professionals to participate. However, this in combination with a lack of experience and the great impetus for work could expose them to greater risks. So, could this conduct by society and the government be seen as ethical? (8).

Furthermore, students who are not selected through a scholarship, and volunteer when summoned by a specific municipality, may lack the financial resources required to participate (5). It is not clear which institution will provide such students with the resources needed to ensure their meaningful participation in the initiative. This violates the equality provided for in the 1988 Brazilian Federal constitution (18).

Finally, we point out that the measure “Brazil Counts on Me” was prepared without prior consultation with the competent bodies such as CNS - National Health Council, Federal Nursing Council (COFEN) and The Brazilian Medical Education Association (17, 19, 20). In fact, the CNS itself points out that the use of health students in training on the frontline of care should be a last resort, to be relied on only after all calls for professionals through other mechanisms have been issued. The hierarchy of intervention scenarios is devised according to the potential risk to the health of students, protecting them from “cognitive, psychic, and occupational stress and working hours” (20).

## Final Considerations

We pointed out and discussed some ethical obstacles contained in the Strategic Action “*O Brasil Conta Comigo*” and envisioned an environment of risk and insecurity for students, not only related to their learning, but also in personal, physical, and emotional aspects. Future health professionals must be prepared to face similar situations, given the characteristics of their academic backgrounds. However, it is prudent to assess the urgency of the situation, and to determine if there is a real need to introduce these students to the front lines of the pandemic response effort, despite their lack of adequate experience. It is necessary to ensure that the Higher Education Institutions from which the participating students originate effectively participate in the supervision thereof. Further, the principle of isonomy should be respected regarding students' participation in the “Brazil Counts on Me” initiative.

In order to reformulate the “*O Brasil Conta Comigo”* Program, there is a need to: (1) map the most fragile areas of SUS - which services are most precarious? Which need more manpower? Which have a larger population for analysis? (2) Define goals for the occupation of sectors with an interval of 3 or 6 months, depending on the logistics available (resources, work team, mobility); (3) invest in formal training for professionals already working; (4) only once all of these efforts have been made should the recruitment of students be considered. Such initiatives should be implemented under the strict supervision of Brazilian public higher education institutions, as was done in the case of another federal program, the “Mais Médicos para o Brasil” program. In the latter initiative, higher education institutions were made responsible for doctors who work in primary care, designating competent professionals to provide face-to-face tutoring, guide face-to-face educational activities and provide classes to improve teaching and learning outcomes. In addition, there must be agreement terms which address all of the particularities inherent in the curricular internship, such as the identification of the advisor, the supervisor, and the activities that will be practiced.

Thus, we see the need to develop new studies that assess the impact of the program on the lives of students who participate in this action, especially regarding psychological and professional aspects, as well as situations triggered by the unequal treatment of applicants entering into residency programs.

## Author Contributions

All authors prepared the review, developed the inclusion criteria, selected titles and abstracts, evaluated the quality of the articles included, and wrote the manuscript.

## Conflict of Interest

The authors declare that the research was conducted in the absence of any commercial or financial relationships that could be construed as a potential conflict of interest.
